# A bioceramic scaffold composed of strontium-doped three-dimensional hydroxyapatite whiskers for enhanced bone regeneration in osteoporotic defects

**DOI:** 10.7150/thno.40103

**Published:** 2020-01-01

**Authors:** Rui Zhao, Siyu Chen, Wanlu Zhao, Long Yang, Bo Yuan, Voicu Stefan Ioan, Antoniac Vasile Iulian, Xiao Yang, Xiangdong Zhu, Xingdong Zhang

**Affiliations:** 1 National Engineering Research Center for Biomaterials, Sichuan University, Chengdu, 610064, China.; 2 Department of Analytical Chemistry and Environmental Engineering, University Politehnica of Bucharest, Bucharest 011061, Romania.; 3 Department of Metallic Materials Science, Physical Metallurgy, University Politehnica of Bucharest, Bucharest 060042, Romania.

**Keywords:** calcium phosphate bioceramics, strontium, bone regeneration, osteoporosis, whiskerization.

## Abstract

Reconstruction of osteoporotic bone defects is a clinical problem that continues to inspire the design of new materials.

**Methods**: In this work, bioceramics composed of strontium (Sr)-doped hydroxyapatite (HA) whiskers or pure HA whiskers were successfully fabricated by hydrothermal treatment and respectively named SrWCP and WCP. Both bioceramics had similar three-dimensional (3D) porous structures and mechanical strengths, but the SrWCP bioceramic was capable of releasing Sr under physiological conditions. In an osteoporotic rat metaphyseal femoral bone defect model, both bioceramic scaffolds were implanted, and another group that received WCP plus strontium ranelate drug administration (Sr-Ran+WCP) was studied for comparison.

**Results**: At week 1 post-implantation, osteogenesis coupled blood vessels were found to be more common in the SrWCP and Sr-Ran+WCP groups, with substantial vascular-like structures. After 12 weeks of implantation, comparable to the Sr-Ran+WCP group, the SrWCP group showed induction of more new bone formation within the defect as well as at the implant-bone gap region than that of the WCP group. Both the SrWCP and Sr-Ran+WCP groups yielded a beneficial effect on the surrounding trabecular bone microstructure to resist osteoporosis-induced progressive bone loss. While an abnormally high blood Sr ion concentration was found in the Sr-Ran+WCP group, SrWCP showed little adverse effect.

**Conclusion**: Our results collectively suggest that the SrWCP bioceramic can be a safe bone substitute for the treatment of osteoporotic bone defects, as it promotes local bone regeneration and implant osseointegration to a level that strontium ranelate can achieve.

## Introduction

Osteoporosis is a common degenerative disease characterized by low bone quality and high bone turnover due to a negative remodelling balance in bone absorption and replacement 1. The increasing prevalence of osteoporosis has led to a considerable decline in quality of life in the elderly because of susceptibility to disease-associated bone fractures 2. The use of bone grafts is the standard to treat skeletal fractures 3. For a long time, bone grafting materials were used for bone repair and regeneration, including bioceramics, bioglasses, metals, and natural and synthetic biodegradable polymers 4. In patients with osteoporosis, bone regeneration is insufficient compared to that of healthy individuals, which might result in weak osseointegration and osteogenesis between osteoporotic bone and the substitute under the condition of fracture 5-6. Metallic implants and cements are commonly used for osteoporotic fractures. Whereas metallic implants are used as primary fixation devices, cements are mainly used as reinforcement of metallic hardware. However, these existing synthesized bone grafts for clinical osteoporotic application lack sufficient bioactivity to promote new bone formation 7.

Calcium phosphate (CP) bioceramics, similar to the inorganic component of the bone matrix, are osteoconductive and osteoinductive biodegradable bone grafting materials adopted for a variety of orthopaedic applications 8. However, there are still concerns regarding the inherent brittleness and low toughness of CP bioceramics, which limits their application to fractures/defects in skeletal locations where physical loading is expected 4, 9. To further improve the mechanical and bioactive capacities, our group previously fabricated a mechanically enhanced CP bioceramic with a three-dimensional (3D) whisker structure (WCP) and proposed its potential application as an artificial bone substitute in load-bearing defects 10. An *in vivo* segmental bone defect model of beagle dogs implanted with WCP bioceramics demonstrated excellent biocompatibility and fast osseointegration 11. Moreover, the WCP bioceramics also have the ability to decrease the bone fracture rate and augment local bone regeneration in a rat model of osteoporotic bone defects at an early stage 12. However, in these previous works, we observed severe bone loss during the natural process of osteoporotic bone healing, which significantly undermined the quality of new bone at a later stage 13. These findings suggest that an advanced strategy for osteoporotic bone regenerating bioceramics should not only focus on the direct osteogenesis of the implant within the defect but also a favourable surrounding environment near the defect. Together with these two factors, satisfactory osteointegration and robust recovery in the body can be achieved.

In previous studies, several inorganic ions, such as strontium (Sr), iron (Fe), copper (Cu), silicon (Si), magnesium (Mg), manganese (Mn) and zinc (Zn), were successfully incorporated into CP to improve its osteogenesis and angiogenesis abilities 14-18, which has further widened the applications of CP bioceramics. Among these bioactive ions, Sr has gained much attention in the recent decade for its ability to enhance new bone formation by activating the Ca-sensing receptor (CaSR) while inhibiting bone resorption by preventing receptor activation of nuclear factor kappa beta ligand (RANKL) expression 19-20. Moreover, Sr was also found to promote endothelial cellular proliferation and tubule formation, both characteristics of angiogenic ability 21. Some *in vivo* studies investigated the interfacial behaviour of Sr-incorporated CP bioceramics with cancellous and cortical bone in an osteoporotic animal model and indicated the beneficial effect of Sr-incorporated CP bioceramics on bone mass around the bone-implant interface 17, 22-23. Sr utilized in the form of strontium ranelate (Sr-Ran) has been widely used for the clinical treatment of osteoporosis in postmenopausal women and could reduce the incidence of fractures 24. Strontium ranelate was available on market as an anti-osteoporosis drug since 2004. However, in the United States, this drug was no longer approved by the FDA since 2014. In the United Kingdom, strontium ranelate (brand name Protelos) was not available after 2017. Safety concerns about systematic administration of this drug connected to severe cardiovascular risks. Orally administered Sr will induce an increase in drug concentration in the blood plasma after intake and cannot effectively reach the implant-tissue interface, particularly necrotic or avascular tissues left after surgery 25. Therefore, Sr loaded onto the implants was expected to offer a sustained supplementation of the element at the implant-tissue interface locally, thus directly enabling effective absorption by tissues in the vicinity. In addition, whether the osteogenic effect of the Sr-loaded implant could achieve that of the non-loaded implant with systematic administration of Sr-Ran remains unknown.

In the current study, we designed and fabricated Sr-incorporated whiskered calcium phosphate bioceramics (SrWCP) via a hydrothermal method to achieve an even distribution of Sr. It is hypothesized that the SrWCP bioceramic would have a slow but long-term release of Sr due to its whiskered structure with high crystallinity. We hope that through this strategy, a safer use of Sr can be achieved, improving local bone formation with less systemic impact. The surface morphology, phase composition, pore structure, mechanical properties and degradation behaviour were characterized *in vitro*. Moreover, we established an ovariectomized rat critical-sized femoral bone defect model to assess the *in vivo* performance of SrWCP compared to the performances of WCP alone and WCP with strontium ranelate administration. The angiogenic and osteogenic potentials of SrWCP were evaluated with micro-CT and histological analysis. Furthermore, at the implant-bone interface, the Sr element distribution was characterized, and a nanoindentation test was performed.

## Materials and methods

### Fabrication and modification of the bioceramics

**Figure 1:**
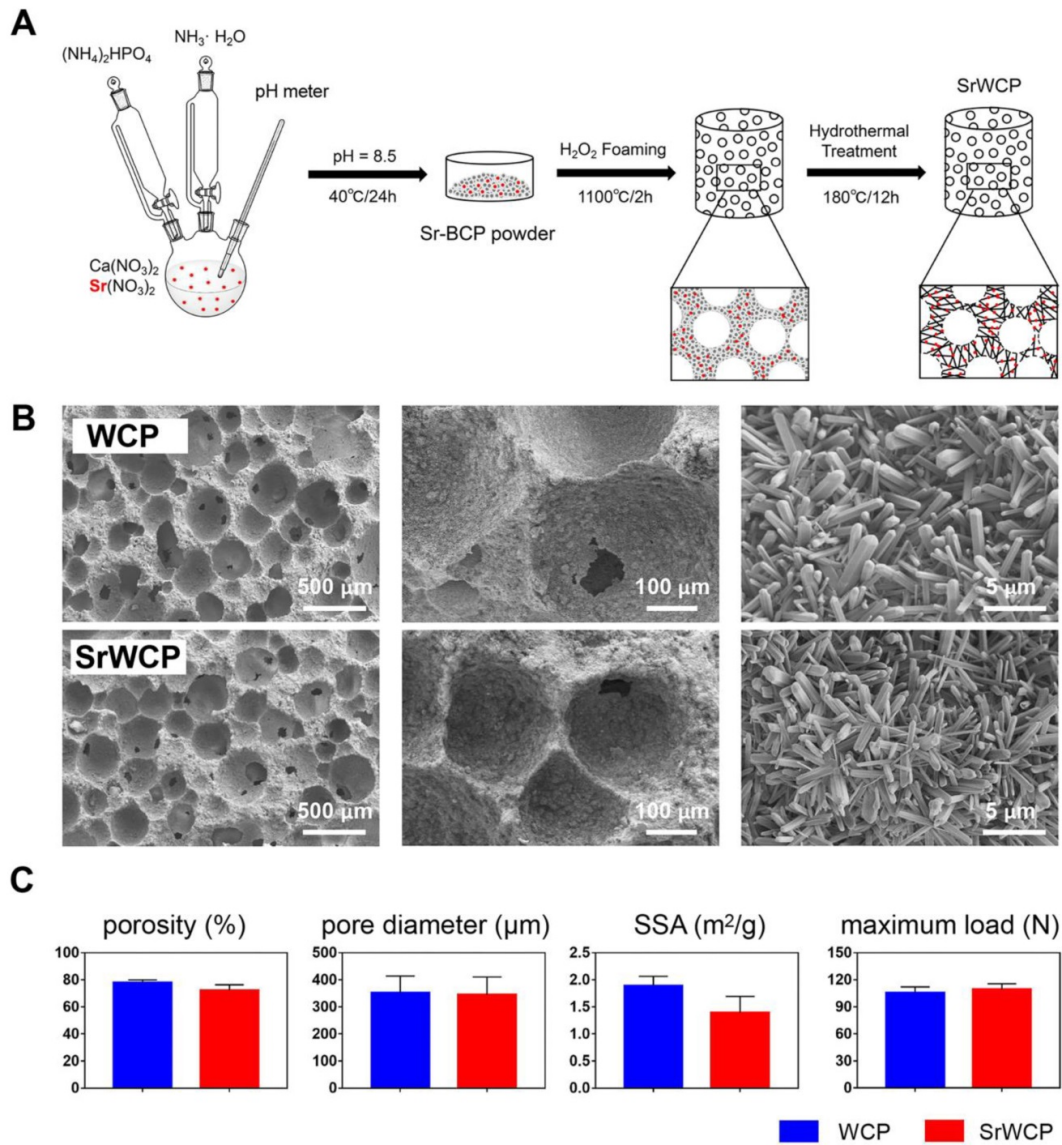
** Synthesis and physiochemical properties of the WCP and SrWCP bioceramics.** (A) The schematic diagram of the fabrication process for the WCP and SrWCP bioceramics. (B) Scanning electron images of the WCP and SrWCP bioceramics. (C) The porosity, mean pore diameter, specific surface area and maximum load of the WCP and SrWCP bioceramics.

A schematic diagram of the fabrication process for WCP and SrWCP is shown in Figure 1A. The amount of reactants used is summarized in Table S1. In brief, 5 L of Ca(NO_3_)_2_ (Sigma-Aldrich, China) and Sr(NO_3_)_2_ (Sigma-Aldrich, China) was added to an equal volume of (NH_4_)_2_HPO_4_ solution (Chengdu Kelong Chemical Reagent Co., Ltd, China) under a continuous stirring rate of 500 rpm and a temperature of 40 °C. The pH of the mixed solution was continuously monitored by a pH meter and adjusted to 8.5 by adding aqueous ammonia. After ageing for 24 h, the solution was washed 3 times with distilled water, dried at 120 °C for 24 h and crushed into powder. The bioceramics were then synthesized by the microwave-assisted H_2_O_2_ foaming method and then sintered at 1100 °C for 2 h at a rate of 5 °C/min increment to 1100 °C. The surface of the two bioceramics was further modified in an aqueous system containing 1 mol/L nitric acid and deionized water (pH = 4.0). The system was transferred into a Teflon-lined stainless-steel autoclave (KH-100 mL, Changyi Co. Ltd, China) at 180 ℃ for 12 h. The obtained WCP and SrWCP bioceramics were washed, dried and shaped into cylinders (diameter 3 × 4 mm^3^) for implantation and into discs (diameter 14 × 2 mm^3^) for *in vitro* tests after sterilization by γ-ray irradiation.

### Characterization of the bioceramics

The surface morphology of the samples was observed by field-emission scanning electron microscopy (SEM, S-4800, Hitachi, Japan), and the elemental mapping of the samples were identified by energy-dispersive X-ray spectrometry (EDS, Oxford, IE250, UK). The mean pore diameter of the bioceramics was measured using SEM images and image analysis software (ImageJ, V1.52). Phase composition of the samples was analyzed by X-ray diffractometry (XRD, Empyrean PANalytical Netherlands). The lattice parameters (a, b and c) of the samples were separately determined by Rietveld refinement and calculated by Jade 6.0 software. The porosity and the specific surface area (SSA) of the samples were performed by mercury intrusion porosimetry (MIP, AutoPore IV 9500, Micromeritics) and surface area analyzer (Gemini VII 2390t, Micromeritics). The samples were investigated using a Fourier transform infrared spectrophotometer (FTIR, Nicolet 6700, Thermo, USA) in the mid-infrared range (400-4000 cm^-1^) using the KBr pellet method. The maximum load of the cylinders (diameter 6 × 10 mm^3^) was studied by a universal testing machine (UTM, AGS-X, Shimadzu, Japan) at a constant loading rate of 1 mm/min. The chemical composition and chemical state of the samples were investigated using X-ray photoelectron spectroscopy (XPS, AXIS Ultra, KRATOS). After implantation, the unstained sections from histological samples were coated with a thin layer of Au and also observed using SEM-EDS to study the elemental distribution at the implant-bone interface.

### Degradation of the bioceramics

To investigate *in vitro* ion release, the WCP and SrWCP bioceramics (diameter 14 × 2 mm^3^) were respectively immersed in citric acid buffer solution (pH = 3.0, solid-liquid ratio = 1:100) and Tris-HCl buffer solution (pH = 7.4, solid-liquid ratio = 1:100) at 37 ℃ for 1, 3 and 7 days. At each time point, the samples were taken out, dried, weighed, and then immersed back to the fresh buffer solution. The amount of Ca, P and Sr ionic concentrations in the medium was examined by an inductively coupled plasma optical emission spectrometer (ICP-OES, SPECTRO ARCOS, Germany) at each prescribed time point. In addition, after complete degradation, the actual Ca, P and Sr content of the WCP and SrWCP bioceramics in the medium was also measured using ICP-OES in citric acid buffer solution.

### Cell Culture

Mouse bone marrow mesenchymal stem cells (MSCs) (Cyagen Biosciences, Guangzhou, China) were used to investigate *in vitro* biocompatibility of the fabricated bioceramics. MSCs were seeded on the WCP and SrWCP bioceramics at 2 × 10^5^ cells per well in 24-well plates coated with Ultra Low Attachment Surface (Corning Inc., Corning, NY). The α-MEM base medium (Gibco, USA) supplemented with 10% standard fetal bovine serum (FBS, Gibco, USA) and 1% penicillin/streptomycin (Gibco, USA) were used. The coculture of cells and bioceramics was maintained at 37 °C under 95% air and 5% CO_2_. As a drug administered control group, strontium ranelate (AK-72916, Ark Pharm, USA) was added into the medium of the WCP coculture (Sr-Ran+WCP) 26. After culturing for 48 h, the samples were stained with fluorescein diacetate (FDA, Sigma, USA) and propidium iodide (PI, Sigma, USA) for five minutes and rinsed with phosphate buffer saline (PBS). Confocal laser scanning microscopy (CLSM, LeicaTCS-SP5, Germany) was then used to observe cell adhesion on the bioceramics of the different groups. Moreover, cell viability was further quantified using a cell counting kit (CCK-8, Dojindo, Japan).

### Animal experiments

All animal experiments were licensed by local animal care committee and carried out in compliance with the Institutional Animal Care and Use Committee (IACUC) of Sichuan University. Following acclimatization, the Sprague-Dawley female rats (12 weeks old, 240 ± 7 g, Chengdu Dashuo Experimental Animal Co. Ltd, China) were subjected to bilateral ovariectomy (OVX) after anesthetization by intraperitoneal injection of nembutal (0.02 mg/kg body weight) as described in our previous study 27. Six weeks after ovariectomy, OVX rats (320 ± 20 g) underwent surgical intervention to establish an intramedullary defect in the distal femur (3 mm in diameter × 4 mm in depth) using a 3.0 mm diameter trephine bur (Fine Science Tools, US). The rats were randomly assigned to three groups according to the implanted materials and drug administration: (1) WCP group: WCP bioceramics implantation with saline administration (n = 12); (2) SrWCP group: SrWCP bioceramics implantation with saline administration (n = 12); (3) Sr-Ran+WCP group: WCP bioceramics implantation with strontium ranelate administration (AK-72916, Ark Pharm, USA, 625 mg/kg/day) (n = 12) 28. After feeding for 1, 8 and 12 weeks, 4 rats of each group were sacrificed using an overdose of pentobarbital. Serum and femur samples were harvested.

### Micro-CT analysis

Microcomputed tomography (micro-CT) system (µCT80, Scanco Medical, Basersdorf, Switzerland) was used to examine the implanted materials at weeks 1, 8 and 12. The scanner was set at an X-ray tube voltage of 70 kVp, a tube current of 114 mA, a pixel matrix of 2048 × 2048 and a resolution of 12 µm per pixel. A global threshold was defined for all scans to extract a physiologically accurate representation of the trabecular bone phase. As previously reported, a gap (100 μm) oriented parallel to the axis of the borehole between the implants and the host bone is our region of interest to study bone integration 29. Quantitative data were calculated from the binarized images through tomographic reconstruction using Mimics Research 17.0 (Materialise Co., Belgium). The volume of new bone formation (nBV, mm^3^) within the drilled hole (nTV, 28.27 mm^3^), the volume of circular new bone formation (cBV, mm^3^) within the circular gap (cTV, 3.90 mm^3^) and the volume of the degraded bioceramics (DV, mm^3^) were then obtained. The new bone substitution rate, regarded as nBV/DV, was previously defined to describe the matching degree between the degradation rate of the bioceramics and the rate of the new bone formation 12. Direct measurements with Scanco analytical software were used to assess trabecular microarchitecture parameters of a cubic volume of interest (VOI = 2 mm^3^) adjacent to the defect, including bone mineral density (BMD, mg HA/ccm), bone volume fraction (BV/TV, %), specific bone surface (BS/BV, mm^-1^), trabecular number (Tb.N, mm^-1^), trabecular thickness (Tb.Th, mm) and trabecular separation (Tb.Sp, mm).

### Histological and immunofluorescence staining

For histological analysis, the harvested femurs after 8- and 12-week implantations were dehydrated in a series of graded concentrations of ethanol solutions from 70% to 100%. After embedding in poly(methylmethacrylate) and cross sectioning (Leica SP1600), the transverse sections (25-µm thickness) were stained with haematoxylin and eosin (H&E) to evaluate bone formation. The area of new bone grew at the interface and inner pores of the implant were analysed separately by IPP 6.0 software. In addition, the samples at week 1 were decalcified in 10% EDTA for 6 weeks and then embedded in paraffin and sectioned mainly for immunofluorescence staining. The expression levels of several vascularization-related proteins and bone remodelling-related proteins were analysed by immunofluorescence staining. Briefly, the sections were incubated with individual primary antibodies (Omnimabs, US) to rat CD31 (OM287804, 1:100), endomucin (Emcn, OM251416, 1:100), tartrate-resistant acid phosphatase (TRAP, OM167217, 1:100) and osteocalcin (OCN, GB11233, 1:100) overnight at 4 °C and then washed three times with PBS. Subsequently, secondary antibodies conjugated with fluorescence were used to cover the sections at room temperature for 50 min while avoiding light. After washing three times with PBS, DAPI solution (G1012, ServiceBio, China) was added dropwise and incubated for 10 min at room temperature to counterstain nuclei. All staining was performed following the manufacturer's protocol. The sections were observed under a confocal microscope and analysed quantitatively by IPP 6.0 software.

### Determination of serum ion concentrations and bone metabolic markers

The blood samples were collected at each time point and centrifuged for 15 minutes at 1,000 × g at 4 °C. To assess the potential systemic side effects, the concentrations of serum strontium and serum calcium were determined by ICP-OES after dilution. Several bone metabolic markers were also evaluated using commercial ELISA kit, including cross-linked C-telopeptide of type I collagen (CTX-I, cat. E0665m, Uscnlife, China), osteocalcin (GE Healthcare Bioscience, Tokyo, Japan) and procollagen I N-terminal peptide (PINP, E90957, Uscnlife, China). The measurement procedures were carried out according to the manufacturer's instructions. A standard curve was made based on the concentration and optical density (OD) value of the standard at the respective wavelength. The concentrations of the markers were calculated according to the standard curve equation.

### Nanoindentation test

Nanoindentation tests were performed using an ultra nanoindenter (UNHT, Switzerland). Briefly, a Berkovich diamond tip was used to indent the surface of samples based on optical microscopic observations. The tip was calibrated prior to the measurement on fused silica to maintain accuracy. Indentations were randomly positioned either inside the remaining materials or the newly formed bone with applied loads up to 10 mN and a loading/unloading rate of 20.00 mN/min. The elastic modulus and hardness for each indentation were calculated from the unloading part of the load-penetration curve using the Oliver-Pharr method. Bone was assumed to be isotropic, with a 0.3 Poisson's ratio. Six indents were produced on each sample (n = 4/group), and the averaged data were used. The average elastic modulus of the newly formed bone (*E*_b_), the hardness of the newly formed bone (*H*_b_), the average elastic modulus of the undegraded material (*E*_m_) and the hardness of the undegraded material (*H*_m_) were then obtained.

### Statistical analysis

All quantitative data were presented as mean ± standard deviation (SD) as indicated and analyzed using one-way analysis of variance (ANOVA) with a Tukey-Kramer post hoc test or Student's t test for two-group comparisons at 95% confidence levels. *p* < 0.05 was considered statistically significant.

## Results

### Characterization of the bioceramics

As mentioned earlier, Figure 1A illustrates the fabrication process of the SrWCP bioceramic via hydrothermal treatment. The Sr substitution level of SrWCP could be facilely tailored by adjusting the initial molar ratio of Sr/(Ca + Sr), and the initial molar ratio of Sr/(Ca + Sr) in SrWCP was 10% (Table S1). According to previous studies, 10% incorporation of Sr could promote the expression of osteogenic genes and bone regeneration 22-23. The fabricated SrWCP and WCP bioceramics are highly porous with macropores, and the macropore size and interconnected pore size were approximately 300 - 450 μm and ~ 100 μm, respectively (Figure 1B). An enlarged view shows that the surfaces of the two bioceramics were composed of 3D micro-whiskers. SEM images demonstrate that whiskers (microrods) with diameters of approximately 500 nm and lengths up to 10 μm were distributed quite uniformly in the SrWCP and WCP bioceramics. The results indicated that incorporating Sr into WCP exerted little influence on the surface structure of the bioceramics. In addition, there are no significant differences in the porosity, mean pore diameter, specific surface area and maximum load between the WCP and SrWCP bioceramics (Figure 1C).

**Figure 2:**
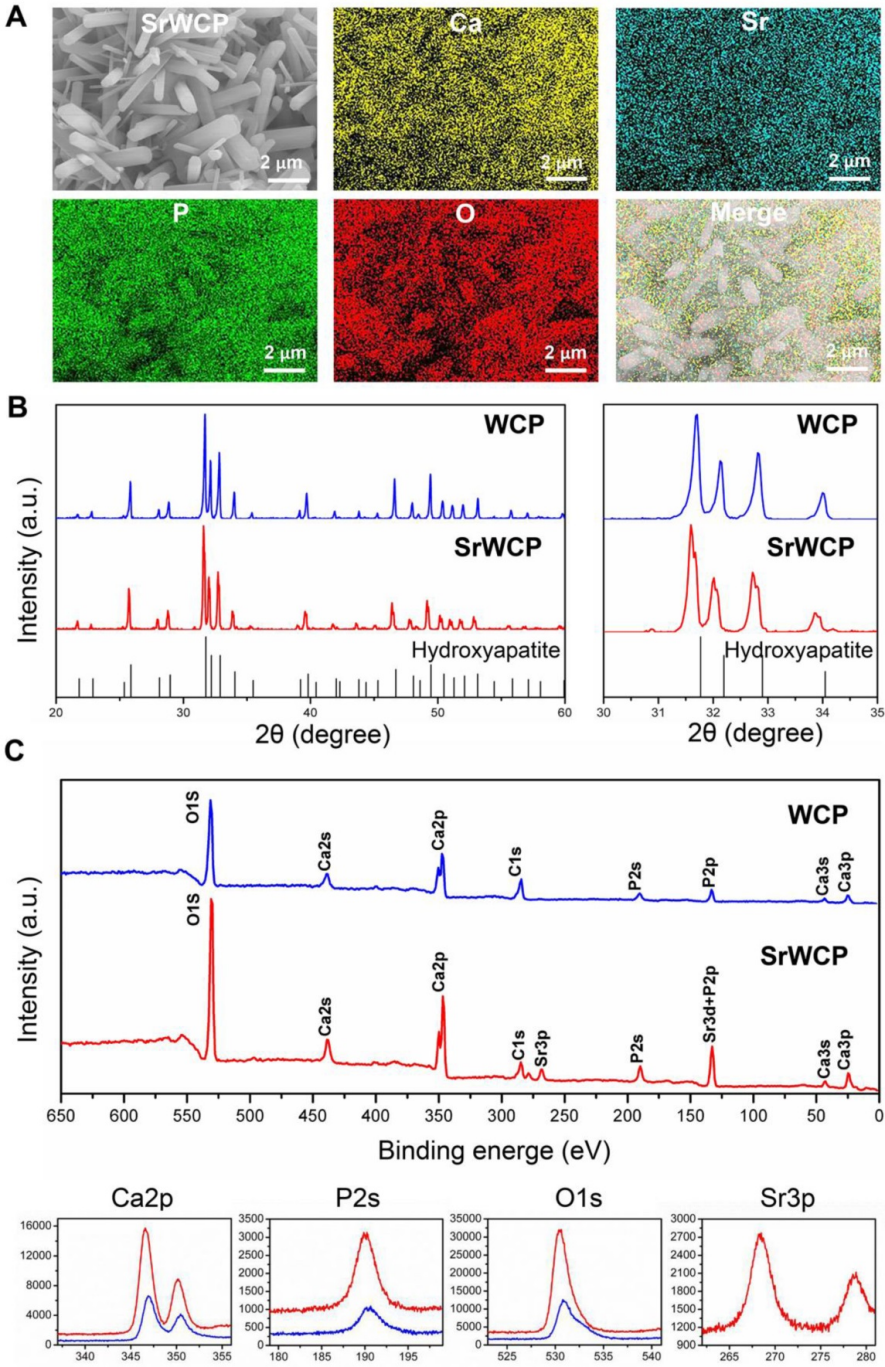
** Phase composition analysis of the WCP and SrWCP bioceramics.** (A) EDS mapping for the major elements of the SrWCP bioceramics. (B) XRD spectra of the WCP and SrWCP bioceramics. Standard spectra of hydroxyapatite was provided below. (C) Comparison XPS spectra (including characteristic peaks of Ca2p, P2s, O1s and Sr3p) of the WCP and SrWCP bioceramics.

Elemental mapping of SrWCP confirmed the presence and homogeneous distribution of Sr and other elements compared to WCP (Figure 2A). The Sr/(Ca + Sr) molar ratio of SrWCP is approximately 11.12% (Figure S1). According to the XRD patterns (Figure 2B), the hydroxyapatite phase is identified in two bioceramics with no other calcium phosphate impurity. The diffraction peaks of SrWCP shift to a slightly smaller 2θ angle (~ 0.178°) compared to that of WCP (Table S2), in agreement with the data previously reported 30. The FTIR spectra of the samples are shown in Figure S2. The spectra of WCP and SrWCP presented similar characteristic bands. Bands at 962 and 1040 cm^-1^ correspond to P-O stretching 15. The actual molar ratio of Sr substitution for Ca and the (Ca + Sr)/P in the bioceramics were further calculated by ICP-OES. The quantitative elemental analysis indicated that SrWCP was obtained with Sr replacement of 10.51% and that the (Ca + Sr)/P ratio was in the generally accepted range of 1.50-1.70. The chemical state of the elements in the bioceramics and Sr distribution along the SrWCP was analysed by XPS (Figure 2C). The characteristic peaks of Ca2s, Ca2p, Ca3s, Ca3p, P2s, P2p and O1s were detected in both WCP and SrWCP bioceramics. Additional peaks of Sr3p and Sr3d indicated the presence of Sr on the surface of the SrWCP bioceramic. The envelopes of the P2p and Sr3d peaks at approximately 133 eV overlapped due to their proximity. Consistent with previous studies, Sr incorporation increased the binding energies of Ca2p, P2s and O1s 31-32.

### Ion release from the bioceramics and its influence on cocultured cells

**Figure 3:**
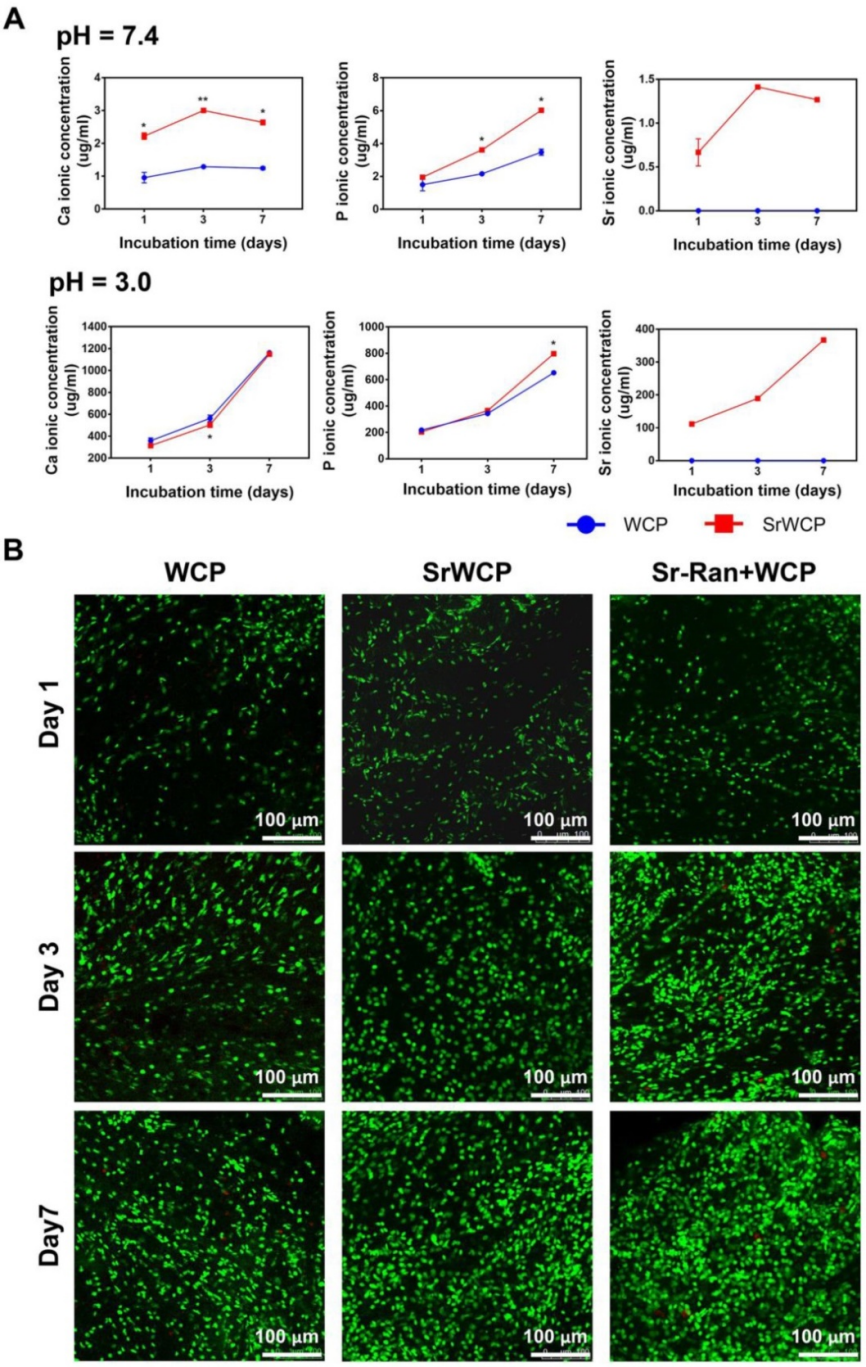
***In vitro* degradation rates of the WCP and SrWCP bioceramics and the viability of cells cultured on the WCP and SrWCP bioceramics.** (A) Time-dependent change of Ca, P and Sr ionic concentrations released from the WCP and SrWCP bioceramics after immersion into in Tris-HCl buffer solution (pH = 7.4) and citric acid buffer solution (pH = 3.0): *p < 0.05 vs the WCP group; **p < 0.01 vs the WCP group. (B) FDA/PI staining of MSCs growth on the different bioceramics at days 1, 3 and 7.

Tris-HCl (pH = 7.4) buffer solution and citric acid buffer solution (pH = 3.0) were used to evaluate the degradation rate of the WCP and SrWCP bioceramics (Figure 3A). With prolonged degradation time, little morphological change can be observed on the surface of SrWCP and WCP besides a few debris (Figure S3). Quantitative analysis revealed that the Ca and P ionic concentrations of the SrWCP group were significantly higher than those of the WCP group in the Tris-HCl solution at days 3 and 7. In citric acid degradation solution, the P ion released from the SrWCP bioceramic was also significantly more than that of WCP at day 7. However, the Ca ion concentration in the SrWCP bioceramic citric acid solution was significantly lower than that in the WCP at day 3. Moreover, continuous Sr released from the SrWCP bioceramic was monitored in the two degradation solutions at each time point. The Sr ion concentration in the citric acid solution was 132-fold higher than that in the Tris-HCl solution due to the intense degradation in the acidic environment. In addition, in the Tris-HCl degradation solution, the WCP group showed a significantly higher Ca/P molar ratio than the SrWCP group. However, the Ca/P molar ratio in the citric acid degradation solution of the SrWCP group was significantly higher than that of the WCP group at days 3 and 7.

To assess the influence of ion release on the viability of MSCs cocultured with different bioceramics, FDA/PI staining and CCK-8 assays were performed (Figure 3B and Figure S4). Confocal observation revealed that a great number of live cells adhered to the material surface, and the cell density increased with culture time in all groups. Compared to the WCP group, more cells were observed in the SrWCP and Sr-Ran+WCP groups after 3 days of culture. At day 7, MSCs cultured on the surface of the bioceramics in the SrWCP and Sr-Ran+WCP groups tended to spread moderately and presented polygonal spreading, while the MSCs on the surface of the WCP bioceramics presented spherical shapes. As determined by the CCK-8 assay, cell proliferation occurred for all three groups across the period examined. The cell viability of MSCs in the SrWCP and Sr-Ran+WCP groups was significantly higher than that in the WCP group at days 3 and 7. No significant differences in cell viability were detected between the SrWCP and Sr-Ran+WCP groups.

### Weight changes and serum analysis

**Figure 4:**
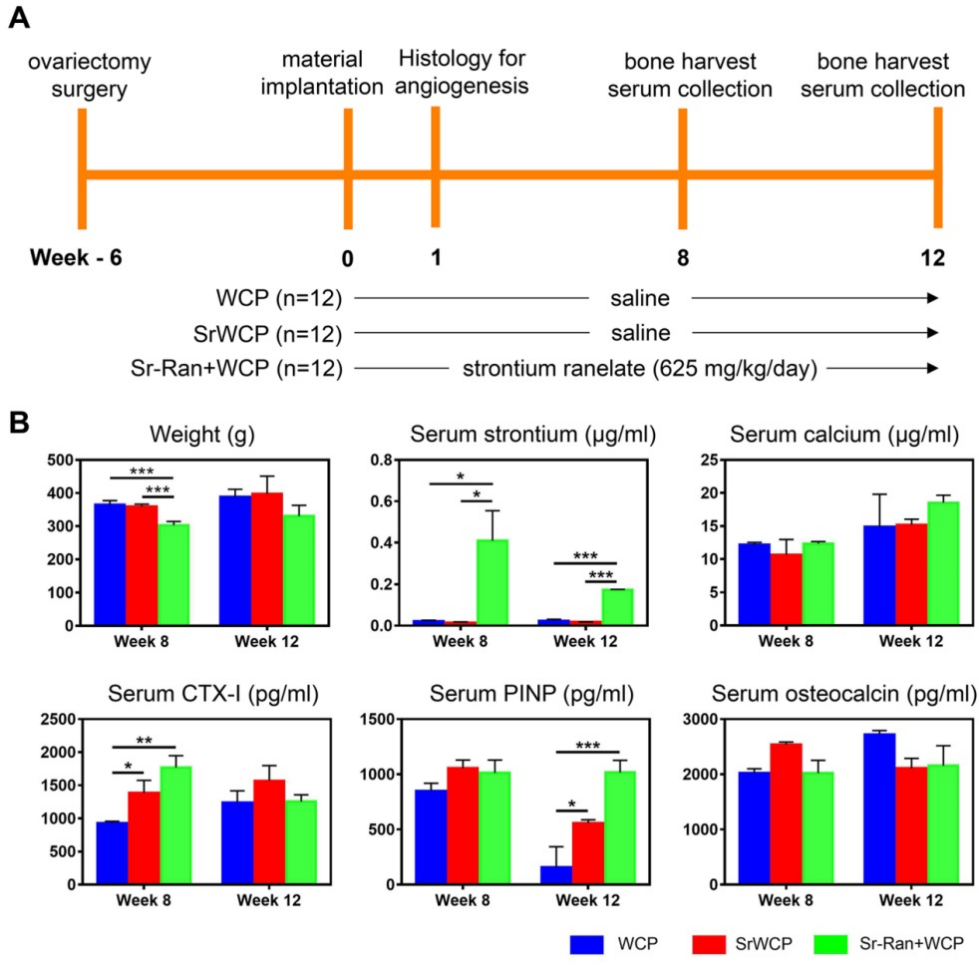
***In vivo* assessment of the WCP and SrWCP bioceramics.** (A) The timeline of the study to evaluate the WCP and SrWCP bioceramics with Sprague-Dawley rats. (B) Time-dependent changes of the body weights, serum ion concentrations (strontium, calcium) and serum levels of the bone formation (PINP, osteocalcin) and resorption (CTX-I) biomarkers (*p < 0.05; **p < 0.01; ***p < 0.001).

Figure 4A shows the timeline and arrangement of the *in vivo* experiments. The body weight changes and serum analysis of different groups at weeks 8 and 12 are shown in Figure 4B. There was an overall upward trend in body weight for all groups. The body weight of the Sr-Ran+WCP group was significantly lower than those of the WCP and SrWCP groups at week 8, while no significant difference in average weight among the groups was observed at week 12. Serum analysis indicated that the WCP and SrWCP groups had a similar serum strontium concentration that was almost undetectable, which is normal. However, the Sr-Ran+WCP group had 22-fold and 8-fold higher serum strontium concentrations than normal at weeks 8 and 12, respectively. There was no significant difference in the serum calcium concentration among the different groups. It is interesting to note that the SrWCP and Sr-Ran+WCP groups showed significantly higher levels of CTX-I than in the WCP group at week 8. The serum PINP level of the SrWCP and Sr-Ran+WCP groups was also significantly higher than that of the WCP group at week 12. For serum osteocalcin, no significant difference was detected among all groups. Moreover, there was no significant difference between the SrWCP and Sr-Ran+WCP groups in all serum biomarkers measured.

### *In vivo* osteoporotic bone repair evaluated by micro-CT

**Figure 5:**
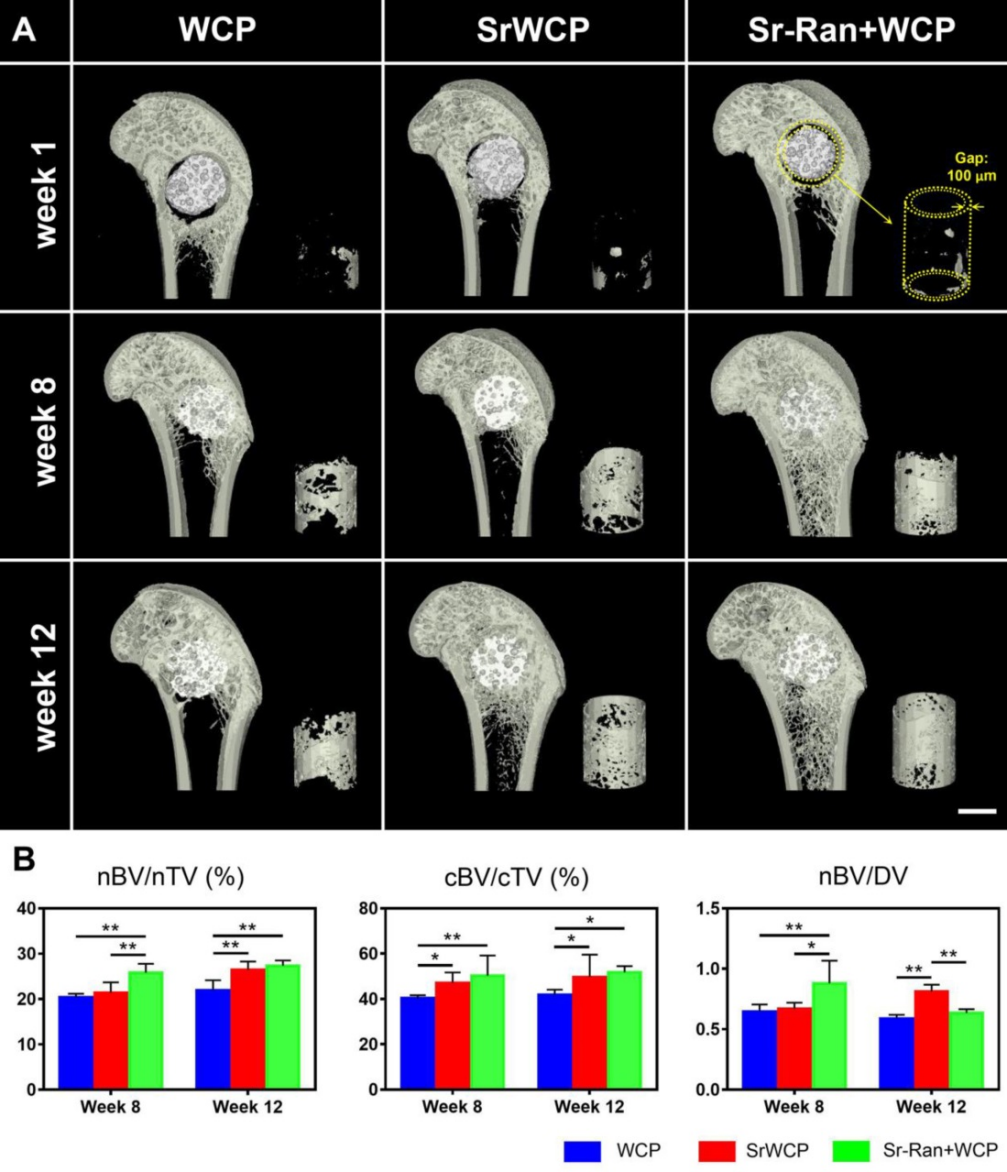
** Micro-CT rendered images and data of the bone formation.** (A) Reconstructed micro-CT images of the coronal sections from the metaphyseal femur at weeks 1, 8 and 12. The materials (white) were embedded in newly formed bone (grey) after implantation. Right corner of each sample: 3D reconstructed images of the newly formed bone inside the gap (100 μm) between the implants and host bone (scale bar = 2 mm). (B) Quantitative analysis of micro-CT data: the new bone formation (nBV, mm^3^) within the drilled hole (nTV, mm^3^) and the volume of circular new bone formation (cBV, mm^3^) within the circular gap (cTV, mm^3^). The original volume of each material (MV) was measured before implantation and the volume of the remaining material (RMV) within the bone defect at each time point can be calculated. Thus, the degraded material (DV) is equal to MV minus RMV. The bone ingrowth rate (nBV/nTV), bone-implant osseointegration rate (cBV/cTV) and bone substitution rate (nBV/DV) were then obtained, respectively (*p < 0. 05, **p < 0.01).

Typical coronal sections of the femurs from different groups are reconstructed (Figure 5A). After 1 week of implantation, the gap (100 μm) between the implants and host bone was still observable. At week 8, all cylindrical implants from each group were well integrated with the femoral epiphyseal bone, and the gap virtually disappeared. It seems that more new bone was achieved within the circular gap (yellow cylinder in Figure 5) in the SrWCP and Sr-Ran+WCP groups at weeks 8 and 12. Quantification of new bone revealed that the nBV/nTV of the Sr-Ran+WCP group was significantly higher than that of the WCP and SrWCP groups at week 8 (Figure 5B). The nBV/nTV of the SrWCP group increased more from week 8 to week 12 compared with that of the other groups. At the end week 12, the SrWCP and Sr-Ran+WCP groups showed a significantly higher nBV/nTV compared to that of the WCP group, but no significant difference between these two groups was observed. In comparison with the WCP group, the SrWCP and Sr-Ran+WCP groups conferred significantly higher cBV/cTV inside the circular gap area, and again, no significant difference between these two groups was detected. The new bone substitution rate, defined as nBV/DV, of the Sr-Ran+WCP group was significantly higher than that of the WCP and SrWCP groups after 8 weeks of implantation. However, the SrWCP group had a significantly higher nBV/DV than that of the WCP and Sr-Ran+WCP groups at week 12. Compared to WCP, the degradation rate of the SrWCP bioceramics matched better with new bone tissue ingrowth at a longer time.

**Figure 6:**
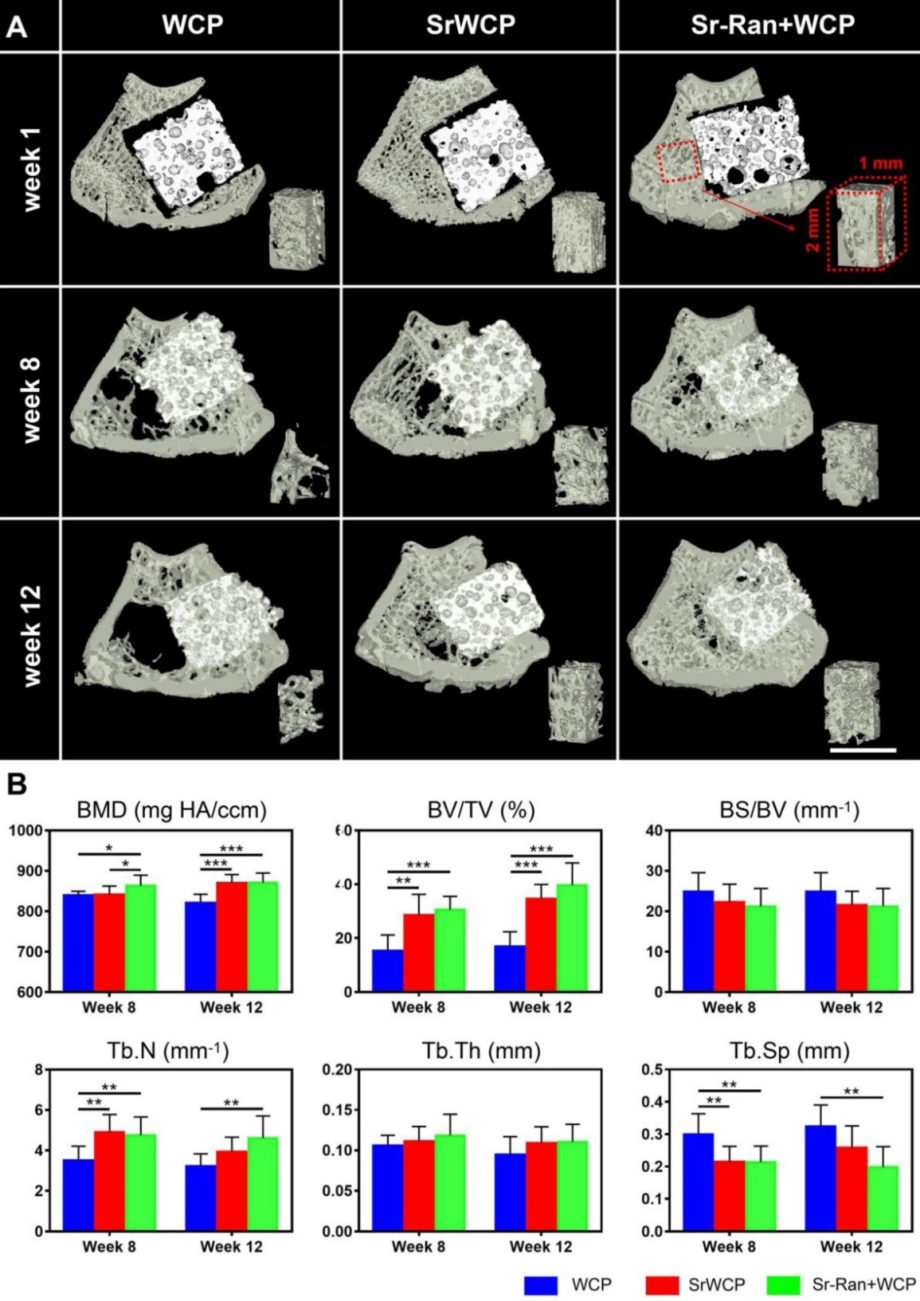
** Evaluation for the effect of the implantation on the trabecular bone adjacent to the implants.** (A) The reconstruction of the transverse sections from the femur samples and the trabecular bone of a cubic volume of interest (VOI) adjacent to the defect (scale bar = 2 mm). (B) Trabecular microarchitecture parameters of VOI, including bone mineral density (BMD, mg HA/ccm), bone volume fraction (BV/TV, %), specific bone surface (BS/BV, mm^-1^), trabecular number (Tb.N, mm^-1^), trabecular thickness (Tb.Th, mm) and trabecular separation (Tb.Sp, mm) (*p < 0.05, ** *p* < 0.01).

The transverse sections of 3D reconstructed micro-CT scanning are further used to evaluate the effect of implantation on trabecular bone adjacent to the implants (Figure 6A). At weeks 8 and 12, the WCP group specifically showed a less dense trabecular mesh and a larger hollow bone marrow region, while more bone was observed around the implants in the SrWCP and Sr-Ran+WCP groups. Quantitative analysis further confirmed that the BV/TV of the adjacent bone in the SrWCP and Sr-Ran+WCP groups was significantly higher than that of the WCP group after 8 and 12 weeks of implantation (Figure 6B). The bone BMD of the Sr-Ran+WCP group was significantly higher than that of the other two groups at week 8. However, at week 12, the BMD of the SrWCP group increased dramatically and was comparable to that of the Sr-Ran+WCP group, which was significantly higher than that of the WCP group. Moreover, Tb.N of the SrWCP and Sr-Ran+WCP groups was significantly higher than that of the WCP group at week 8. The Sr-Ran+WCP group showed a significantly higher Tb.N compared to the WCP group at week 12, but no significant difference between the SrWCP and Sr-Ran+WCP groups was observed. The WCP group had a significantly higher Tb.Sp value compared to the values of the SrWCP and Sr-Ran+WCP groups at week 8. The WCP group showed the highest Tb.Sp value throughout the study, revealing sparsely distributed trabeculae in the WCP group compared with those of the other groups. No significant differences in BS/BV and Tb.Th were observed among all groups.

### *In vivo* angiogenesis and osteogenesis at the early stage of implantation

**Figure 7:**
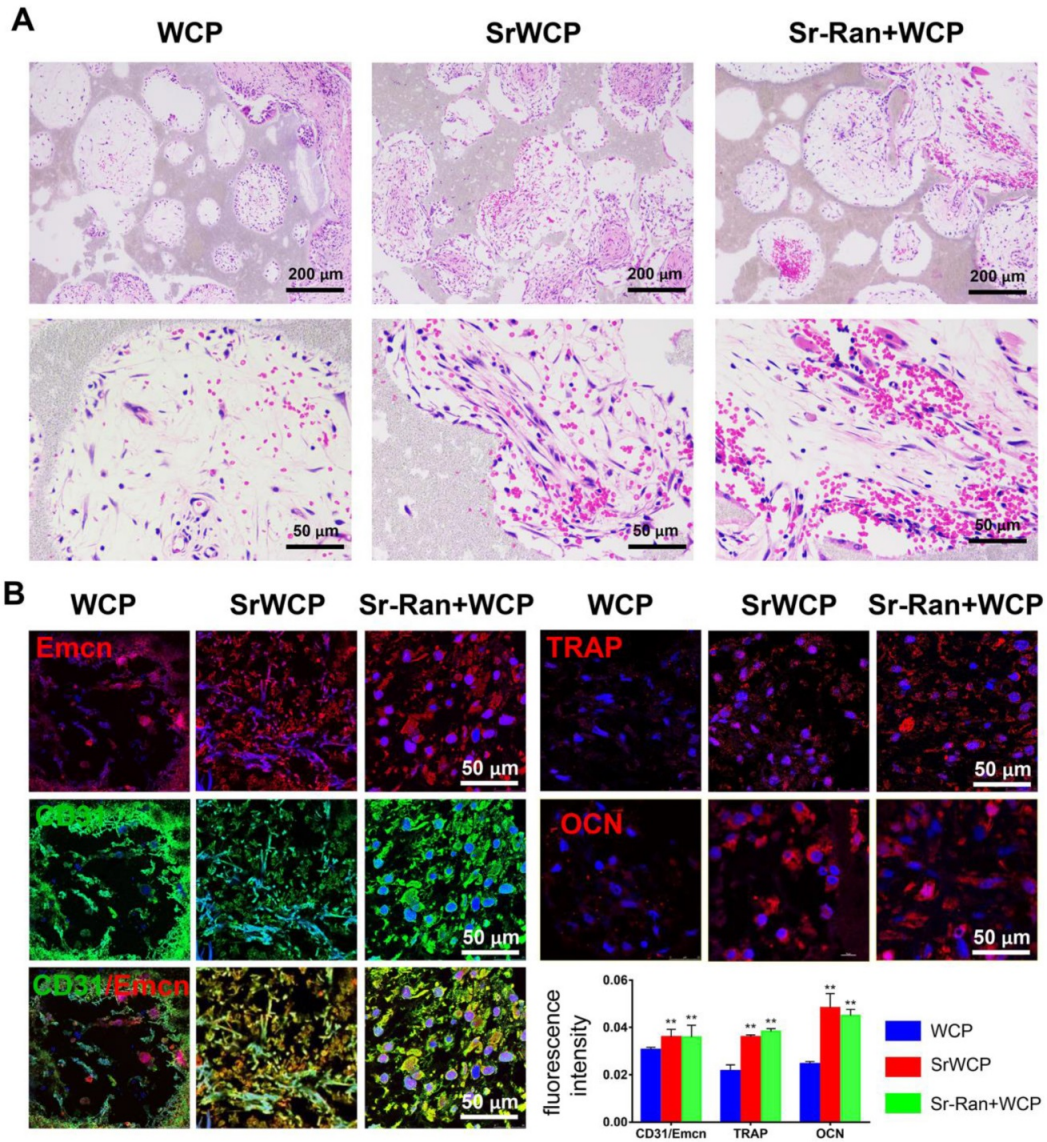
** Evaluation of the *in vivo* angiogenesis, osteogenesis and osteoclastogenesis after implantation for 1 week.** (A) H&E histological images of the bioceramics with the infiltrated tissue: the first row: general view of the section; the second row: magnification of the center area of the bioceramics from each group. (B) Immunofluorescence staining: Green fluorescence indicates active CD31; red fluorescence indicates active Emcn, TRAP and OCN respectively; blue fluorescence indicates PI bound to the nuclei of cells. Quantitative analyses of the mean fluorescence intensity values of CD31, Emcn and TRAP were provided below (*p < 0.05 vs the WCP group; **p < 0.01 vs the WCP group).

Angiogenesis at week 1 after implantation was evaluated by histological analyses. As shown in Figure 7A, H&E staining demonstrated that certain cells in the SrWCP and Sr-Ran+WCP groups had formed vascular-like structures. Under higher magnification of the staining, red blood cells gathered within this structure can be seen. A recent study showed that a specific vessel subtype, the CD31/Emcn vessels, couples angiogenesis and osteogenesis 33. Thus, immunofluorescence staining of these two markers was conducted in the current study. The results show that a larger number of CD31 and Emcn double positive vessels can be observed inside the pores of the SrWCP and Sr-Ran+WCP groups than in the WCP group, indicating the formation of new blood vessels for bone formation (Figure 7B). The fluorescence density of CD31/Emcn vessels was significantly higher in the SrWCP and Sr-Ran+WCP groups than that in the WCP group. TRAP staining here is used to investigate osteoclast activity at the early stage of implantation (Figure S5). Compared to the WCP group, more TRAP-positive cells were observed in the SrWCP and Sr-Ran+WCP groups. More multinucleated osteoclasts were observed in the TRAP-positive expression regions of the SrWCP and Sr-Ran+WCP groups. The osteogenic activity of the bioceramics was also evaluated by immunofluorescent staining for the osteogenic marker OCN. OCN was much more highly expressed in the SrWCP and Sr-Ran+WCP groups than that in the WCP group at week 1 post-implantation. Moreover, there was no significant difference between the SrWCP and Sr-Ran+WCP groups for osteogenic marker expression.

### Histological analysis of osteoporotic bone regeneration

**Figure 8:**
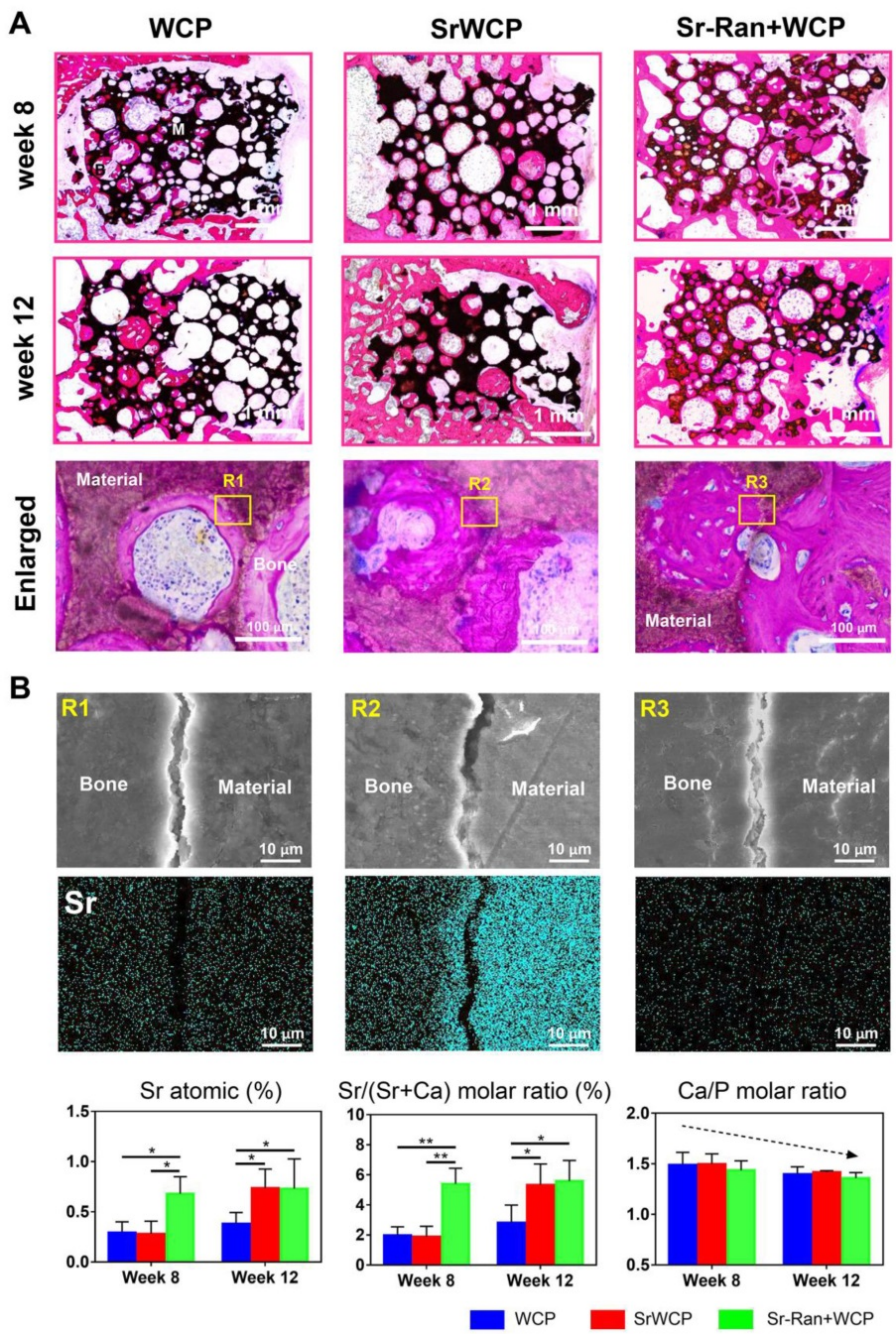
** Histological staining for the bone regeneration and elemental analysis at the bone-bioceramics interface.** (A) Hematoxylin and eosin (H&E) staining of the different groups at week 8 and 12. The magnified images showed osteocyte residence and new bone formation on the wall of the pores. (B) EDS mapping for the major elements at the tissue-implants interface (yellow box in the upper row) and composition determination of the new bone adjacent to the implants (*p < 0.05, **p < 0.01).

H&E staining demonstrated that the SrWCP and Sr-Ran+WCP groups both exhibited tight contact with host bone compared to that of the WCP group (Figure 8A). At week 8, higher amounts of newly calcified bone and extracellular matrix (stained red) in the pores of the bioceramics (stained black) were achieved in the Sr-Ran+WCP group. However, the SrWCP group presented more bone formation at week 12, which was comparable to the Sr-Ran+WCP group. Further quantitative analysis for new bone area was performed based on the H&E-stained sections, and the results are shown in Figure S6. With the prolongation of the implantation time in the body, the amount of newly formed bone of the WCP group had a limited increase, but the amount of newly formed bone increased dramatically with SrWCP. Consistent with the above micro-CT results, new bone in the drilled hole area and gap area of the SrWCP and Sr-Ran+WCP groups was significantly higher than that of the WCP group, and again, no significant difference between these two groups was detected. At a higher magnification, it was observed that more residing osteocytes were distributed on the newly formed bone near the pore wall of the bioceramics. Part of the degrading material was surrounded by new bone tissue, indicating that the degradation progress of the bioceramics was accompanied by the ingrowth of the new bone. In addition, there was less undegraded material in the SrWCP group than in the WCP and Sr-Ran+WCP groups, suggesting that SrWCP had a faster *in vivo* degradation rate, which was consistent with the *in vitro* degradation results.

### Bone-bioceramics boundary analysis

Elemental mapping of the unstained sections at week 12 was used to study the major element distribution at the boundary between the newly formed bone and the materials (Figure 8B). The border between the bioceramics and the bone is partially cracked, and micro-whiskers were extruded into the bone in all groups (Figure S7). Furthermore, the Ca, P, O and Sr elements were homogeneously distributed in the bone tissue adjacent to the implants of all groups. Quantitative analysis revealed that the Sr atomic percentage and Sr/(Sr+Ca) molar ratio of the newly formed bone in the Sr-Ran+WCP group were significantly higher than those in the other groups at week 8. It is important to note that at week 12, there was an observed Sr atomic percentage elevation in the SrWCP group compared to week 8, resulting in significantly higher Sr/(Sr+Ca) molar ratio values, which was comparable to that of the Sr-Ran+WCP group. In addition, the Ca/P molar ratio of all groups was reduced from week 8 to 12 due to the development of osteoporosis.

### Nano-level mechanical properties of the implants and newly formed bone

**Figure 9:**
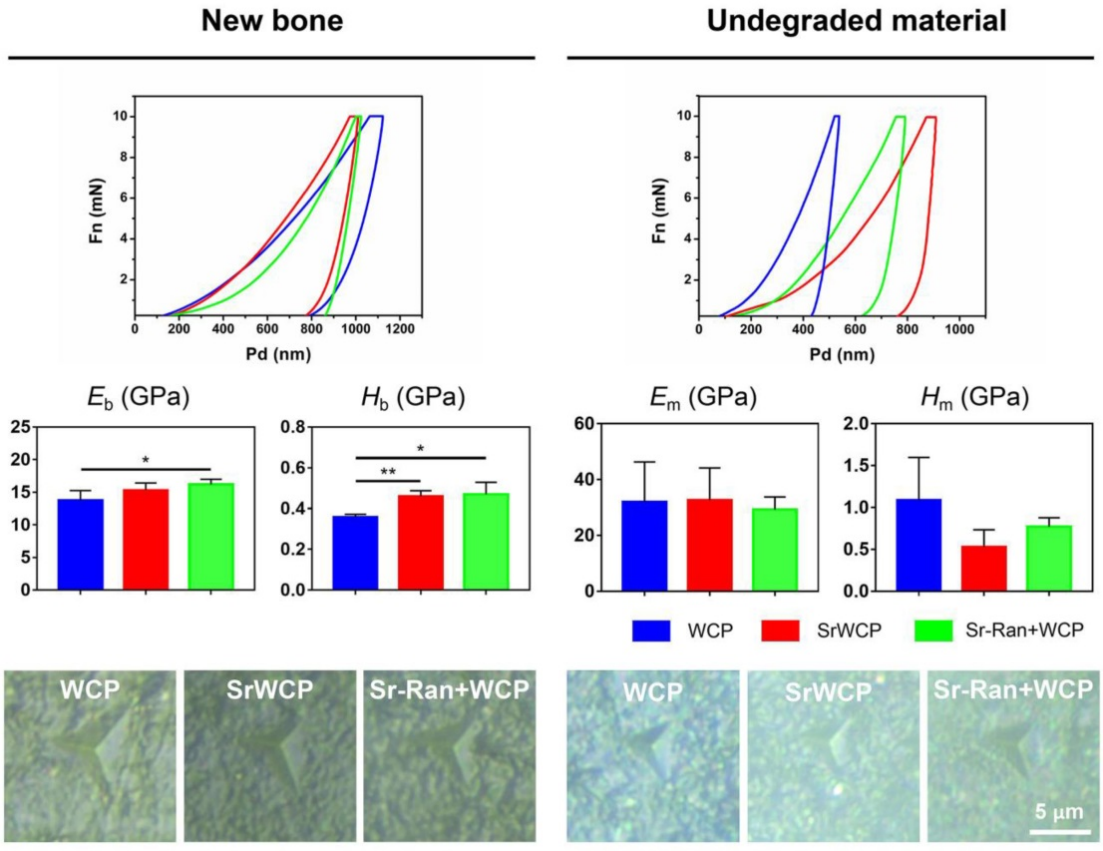
Nanoindentation tests on bone and material: representative loading-unloading curve, average elastic modulus of the newly formed bone (*E*_b_), the hardness of the newly formed bone (*H*_b_), the average elastic modulus of the undegraded material (*E*_m_) and the hardness of the undegraded material (*H*_m_). Optical images of the indentation residues of each group were obtained (*p < 0.05, **p < 0.01). Optical images of the indentation residues of each group were shown below.

To assess the mechanical properties of the newly formed bone and the remaining materials in the healing area of all the groups, nanoindentation was conducted on week 12 bone specimens along the bone transverse axis (Figure 9). Nanoindentations were done with designated position firstly observed under the equipped optical microscope. The boundaries between new bone and the remaining material can be distinguished. The elastic modulus of the newly formed bone under osteoporotic conditions is in a range of 10-18 GPa. The elastic modulus of the remaining materials is in a range of 20-50 GPa. *E*_b_ in the WCP group was significantly lower than that of the Sr-Ran+WCP group. *H*_b_ in the WCP group was greatly lower than that of the SrWCP and Sr-Ran+WCP groups (*p* < 0.05). Moreover, no significant differences were observed in *E*_b_ and *H*_b_ between the SrWCP and the Sr-Ran+WCP groups. There was no statistically significant difference in *E*_m_ and *H*_m_ among all groups. Furthermore, we found that the implanted bioceramics had a much higher *E*_m_ than the neighbouring trabecular bone, while the *H*_m_ of the SrWCP and Sr-Ran+WCP groups was close to the average *H*_b_. It can be reflected in optical images of the indentation residues from each group that the new bone in the SrWCP and Sr-Ran+WCP groups presented a small regular tip indentation residue area (shown in Figure 9), while that of the WCP group showed a relatively larger indentation residue area suggesting a much softer matrix.

## Discussion

Osteoporosis in the ageing population and postmenopausal women often induces pathological fractures 34. Strontium ranelate, a therapeutic for osteoporosis, markedly reduces the risk of fractures by improving bone quality and bone strength in patients 24, 35-36. However, the therapy has its own limitations and adverse effects related to systemic use, such as myocardial infarction, cardiac disorders and cardiac failure 37-38. Thus, local release of Sr by incorporating Sr into biomaterials might be an alternative method applied to the defective osteoporotic bone site. In this study, we developed a Sr-incorporated and mechanically improved whiskered bioceramic for the local release of Sr. Its potential to successfully repair large bone defects and regenerate new bone was demonstrated in an osteoporotic rat model.

The WCP and SrWCP bioceramics were fabricated by hydrothermal reaction and showed an interconnected porous structure. The hydroxyapatite phase was identified in two bioceramics with no other amorphous phase, indicating the complete incorporation of Sr into the bioceramics. It was also shown that Sr was homogeneously distributed on the SrWCP bioceramics. The XPS results further confirmed that Sr incorporation increased the binding energies of Ca2p, P2s and O1s compared to those of WCP, while additional peaks of Sr3p and Sr3d were also detected. No significant differences in the porosity, mean pore diameter, specific surface area or maximum load were detected between the WCP and SrWCP bioceramics. These results imply that the incorporation of Sr would not compromise the superior physical properties of the WCP, which can be considered as a univariate analysis on the effect of Sr release. In addition, the release of Sr from the SrWCP bioceramics was slow, and the concentration did not change much within 7 days in Tris-HCl degradation solution (pH = 7.4). In the citric acid degradation solution (pH = 3.0), the SrWCP bioceramic would degrade rapidly, and a large amount of Sr was released. Whether in the mild extracellular environment or in the acidic environment of intracellular organelles, the release of Sr from SrWCP can be achieved to different degrees.

*In vitro* experiments showed that SrWCP did not inhibit cell viability but promoted MSCs proliferation, which suggested that the SrWCP bioceramic is a biocompatible material. We then used an osteoporotic rat model with critical-sized bone defects to evaluate the bone regeneration ability of the SrWCP bioceramics. First, we studied the levels of bone formation biomarkers, bone resorption biomarkers and different ion concentrations in animal serum. The higher contents of PINP and osteocalcin in the serum were signs of faster bone formation, while the higher content of serum CTX-I was a sign of faster bone resorption 39-40. The animals in the SrWCP and Sr-Ran+WCP groups showed significantly higher levels of PINP and CTX-I in the serum than levels in the WCP group, suggesting that both the systemic administration and local release of Sr increased the bone remodelling turnover rate. We found that the strontium concentration in the blood of the SrWCP group was maintained at a very low level similar to that of the WCP group, which suggested a slim chance of Sr side effects on overall health. However, the serum strontium concentration of the Sr-Ran+WCP group was more than 8-fold higher than that of the other two groups throughout the study, indicating a potential risk of drug administration 28.

The promotion of angiogenesis at an early stage of implantation could significantly enhance osteogenesis 41-42. Several studies demonstrated that Sr released from bioceramics can stimulate angiogenesis by increasing cellular pro-angiogenic cytokine secretion 43-44. Our experiments also showed that in the SrWCP and Sr-Ran+WCP groups, vascular-like structures were formed inside pores in the centre region of the bioceramics one week post-surgery. A specific capillary endothelium type was recently identified in a murine skeletal system that is strongly positive for both CD31 and Emcn 33. It possesses signalling properties that support bone formation and regeneration. This new capillary type is able to mediate perivascular osteoprogenitor differentiation and couple angiogenesis to osteogenesis 45-46. In the current study, we found that both the SrWCP and Sr-Ran+WCP groups exhibited more CD31/Emcn-positive vessels than did the WCP group, indicating that supplementation with Sr played a role in enhancing early angiogenesis related to osteogenesis. In addition, there was an increase in the number of TRAP-positive cells in the SrWCP and Sr-Ran+WCP groups compared to the number in the WCP group, suggesting activated osteoclastogenesis. This result was consistent with the finding of Thormann et al., which showed elevated TRAP expression in the Sr-modified calcium phosphate cement-implanted group in the metaphyseal defect of ovariectomized rats 47. Moreover, cells in the SrWCP and Sr-Ran+WCP groups tended to secrete more OCN protein than those in the WCP group, which has been considered as an important factor in osteogenic differentiation 48. Consistent with the serum biomarker results, the animals in the SrWCP and Sr-Ran+WCP groups had a higher bone turnover rate at the cellular level in the early stage of implantation.

Micro-CT analysis further revealed that the SrWCP and Sr-Ran+WCP groups had more new bone formation within the defective area than the WCP group at week 12, indicating an osteogenic effect with Sr supplementation. Specifically, we found that more new bone was formed in the gap area (100 μm) between the implants and host bone tissue of the SrWCP and Sr-Ran+WCP groups. The newly formed bone in the gap area can effectively bridge host bone and implants, and thus, better osseointegration at the bone-to-implant interface can be achieved. This finding was in line with Andersen et al., who previously discovered that the local delivery of strontium from surface functionalized titanium implants could enhance bone-to-implant contact 49. Furthermore, the material-bone substitution rate, defined as nBV/DV, of the SrWCP group was significantly higher than that of the other groups after 12 weeks of implantation. In other words, upon degrading the same volume of bioceramics, more newly formed bone was induced in the SrWCP bioceramic. Although both the WCP and SrWCP bioceramics were hydroxyapatite ceramics, the SrWCP bioceramics had a faster degradation rate than that of WCP, which was in accordance with previous studies 32. The faster degradation rate of the SrWCP bioceramic matched the ingrowth of new bone in the defected area.

At the end of the study, we observed that the WCP group showed a less dense trabecular structure and a larger hollow bone marrow cavity towards the diaphyseal region, which was consistent with studies that reported irreversible microarchitectural changes within the first 8 to 12 weeks after ovariectomy 50-51. In contrast, the SrWCP and Sr-Ran+WCP groups revealed beneficial effects not only on the metaphyseal bone tissue formation around the implants but also on the trabecular structure maintenance towards the diaphyseal region, with prolonged implantation duration. It was further confirmed that the SrWCP and Sr-Ran+WCP groups had significantly higher BV/TV, Tb.Th and Tb.N of the host bone surrounding the implants compared to the WCP group. These results indicated that both oral administration and local supplementation of Sr could have beneficial effects on preserving bone microarchitecture and volume at the defect region as well as the surrounding host bone. Sr supplementation can effectively inhibit bone loss around the implant material, thereby improving osseointegration. Histological staining demonstrated that the SrWCP and Sr-Ran+WCP groups underwent successful defect regeneration at week 12. The SrWCP and Sr-Ran+WCP groups had more new bone formation at the interface after 8 and 12 weeks compared to that of WCP, which was consistent with the micro-CT results and indicated better osteointegration. We also found that the bone adjacent to implants of the SrWCP group exhibited a significantly higher Sr molar ratio and Sr/(Ca+Sr) than that of WCP at week 12, which can reach the level of systemic administration of Sr-Ran. The findings demonstrated that the local release of Sr from the SrWCP bioceramics into the defective region was sufficient to yield a satisfactory bone regeneration effect, which was comparable to that of the Sr-Ran administration.

Finally, a nanoindentation test was performed to investigate the mechanical properties of the newly formed bone and the remaining materials within the defect area. Previous studies indicated that the intrinsic quality of trabecular bone was undermined by the progression of osteoporosis, and strontium ranelate treatment restored the hardness and toughness of the bone 28, 52. In our previous work using the nanoindentation technique on osteoporotic bone, we discovered that the average elastic modulus of the bony matrix reflected its collagen content, while the hardness was related to the hard mineral content 40, 53-54. The significantly higher *E*_b_ of the Sr-Ran+WCP group compared to WCP may be the result of a modification of the type-1 collagen bone matrix. It was reported that strontium ranelate could stimulate the synthesis of bone collagen *in vitro* 55. Based on histological and EDS results, we speculate that the observed significantly higher *H*_b_ of the SrWCP and Sr-Ran+WCP groups can be attributed to a more mineralized new bone matrix with incorporation of Sr.

## Conclusions

In this study, we developed a Sr-incorporated whiskered calcium phosphate bioceramic and demonstrated its potential to successfully repair large bone defects under osteoporotic conditions. The SrWCP bioceramic was fabricated by hydrothermal reaction and achieved a sustained release of Sr *in vitro*. At an early stage of implantation, enhanced angiogenesis coupled with a fast bone turnover rate was found in both the SrWCP and Sr-Ran+WCP groups. After 12 weeks of implantation, significantly higher new bone formation within the defected area as well as at the implant-bone interface was achieved in the SrWCP and Sr-Ran+WCP groups compared to that of the WCP group. Furthermore, the released Sr from SrWCP further yielded a beneficial effect on the osteoporotic bone quality of the surrounding host bone, which was comparable to that of the Sr-Ran drug treatment. It is interesting to note that the animals from the WCP and SrWCP groups had a similar low serum strontium concentration, whereas the Sr-Ran+WCP group showed a more than 8-fold higher serum strontium concentration than normal, indicating a potential risk. Our results showed that the SrWCP bioceramic can be a promising and safe bone substitute for the treatment of osteoporotic bone defects.

## Supplementary Material

Supplementary figures and tables.

## Abbreviations

BCP: biphasic calcium phosphate
WCP: whiskered calcium phosphate
PINP: a marker of bone formation
CTX-I: a marker of bone resorption
nBV: new bone volume within the drilled hole
cBV: new bone volume within the gap
BMD: bone mineral density
BV/TV: bone volume fraction
BS/BV: specific bone surface
Tb.N: trabecular number
Tb.Th: trabecular thickness
Tb.Sp: trabecular separation
*E*_b_: elastic modulus of the bone
*H*_b_: hardness of the bone
*E*_m_: elastic modulus of the material
*H*_m_: hardness of the material
