# Supplementary material for: A bioceramic scaffold composed of strontium-doped three-dimensional hydroxyapatite whiskers for enhanced bone regeneration in osteoporotic defects
Source: Theranostics. 2020 Jan 1;10(4):1572–89. doi: 10.7150/thno.40103 (PMC6993240; doi:10.7150/thno.40103)
Supplement: Supplementary file 1 — Supplementary figures and tables. [file thnov10p1572s1.pdf]

Supplementary Information

A bioceramic scaffold composed of strontium-doped three-dimensional hydroxyapatite whiskers for enhanced bone regeneration in osteoporotic defects

Rui Zhao[1], Siyu Chen[1], Wanlu Zhao[1], Long Yang[1], Bo Yuan[1], Voicu Stefan Ioan[2], Antoniac Vasile Iulian[3], Xiao Yang[1,*], Xiangdong Zhu[1,*], Xingdong Zhang[1]

[1] National Engineering Research Center for Biomaterials, Sichuan University, Chengdu, 610064, China

[2] Department of Analytical Chemistry and Environmental Engineering, University Politehnica of Bucharest, Bucharest 011061, Romania

[3] Department of Metallic Materials Science, Physical Metallurgy, University Politehnica of Bucharest, Bucharest 060042, Romania

*Corresponding Authors

Tel.: 86-28-85417654;

Fax: 86-28-85410246

E-mail address: xiaoyang114@scu.edu.cn (Xiao Yang) and zxd7303@163.com (Xiangdong Zhu)

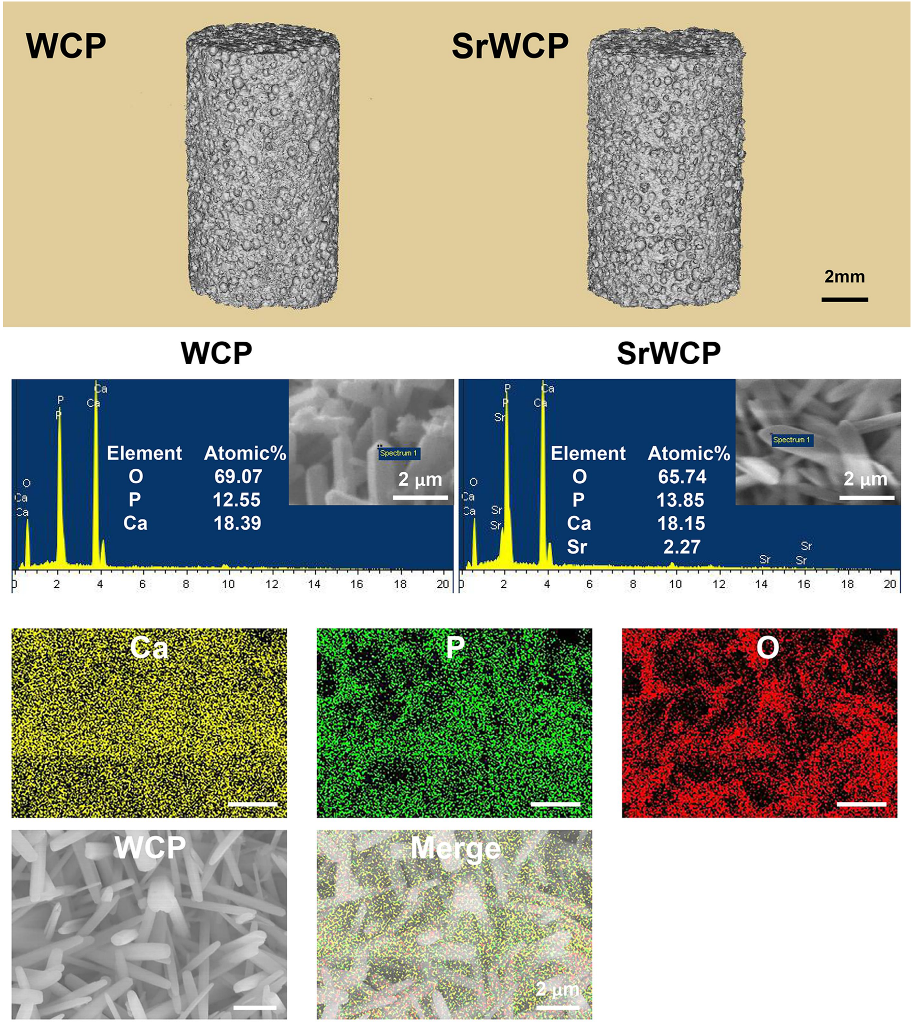

Figure S1. The three-dimensional reconstructed micro-CT images of the WCP and SrWCP scaffolds (Φ 6 × 10 mm^3), EDS spectra of the WCP and SrWCP bioceramics, and EDS mapping for the major elements (Ca, P and O) of the WCP bioceramics.

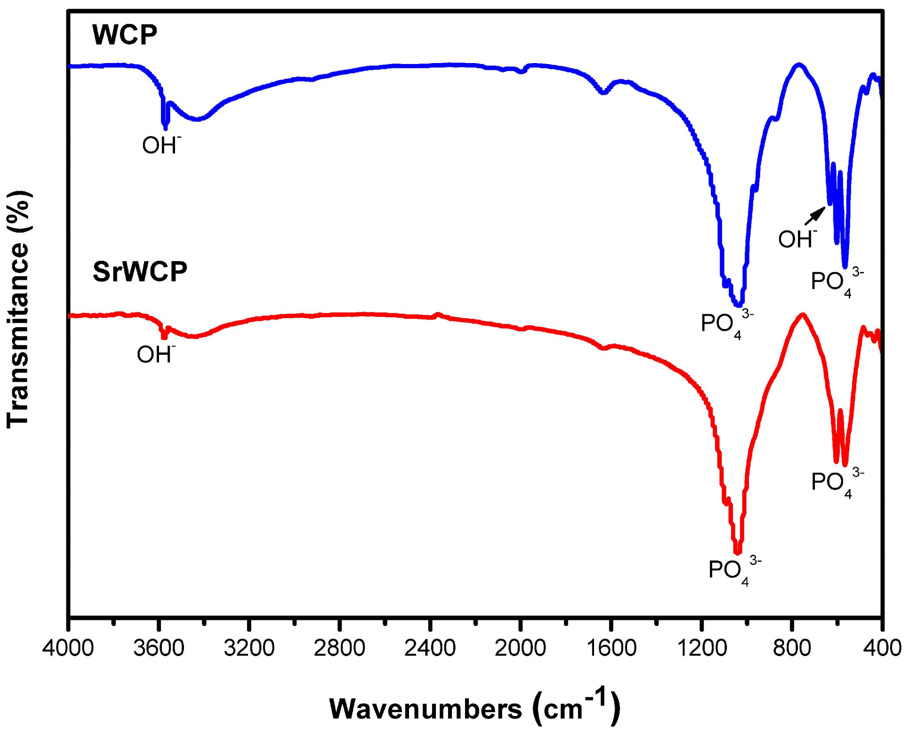

Figure S2. FTIR spectra of the WCP and SrWCP bioceramics.

Table S1

The amounts of the reactants for the synthesis of the WCP and SrWCP powders and theoretical molar ratio of Sr substitution for Ca in powders.

Samples	$Sr(NO_3)_2$ (mM)	$Ca(NO_3)_2$ (mM)	$(NH_4)_2HPO_4$ (mM)	Theoretical molar ratio of Sr substitution for Ca (%)
WCP	0	770	500	0
SrWCP	77	693	500	10

Table S2

Crystallinity, lattice constants, 2θ (°) for (2 1 1) diffraction and chemical composition of the WCP and SrWCP bioceramics.

Samples	Crystallinity (%)	Lattice constants[a]		2θ (°) for (2 1 1) reflection[a]	Chemical composition	
		a (Å)	c (Å)		Ca replacement by Sr (mol. %)	(Ca + Sr)/P molar ratio
WCP	98.72	9.4375	6.8829	31.700	0	1.60
SrWCP	98.99	9.4708	6.9186	31.622	10.51	1.58

[a] Hydroxyapatite (JCPDS 090432, a=b=9.418 Å, c=6.884 Å, 2θ for (2 1 1) diffraction=31.86°).

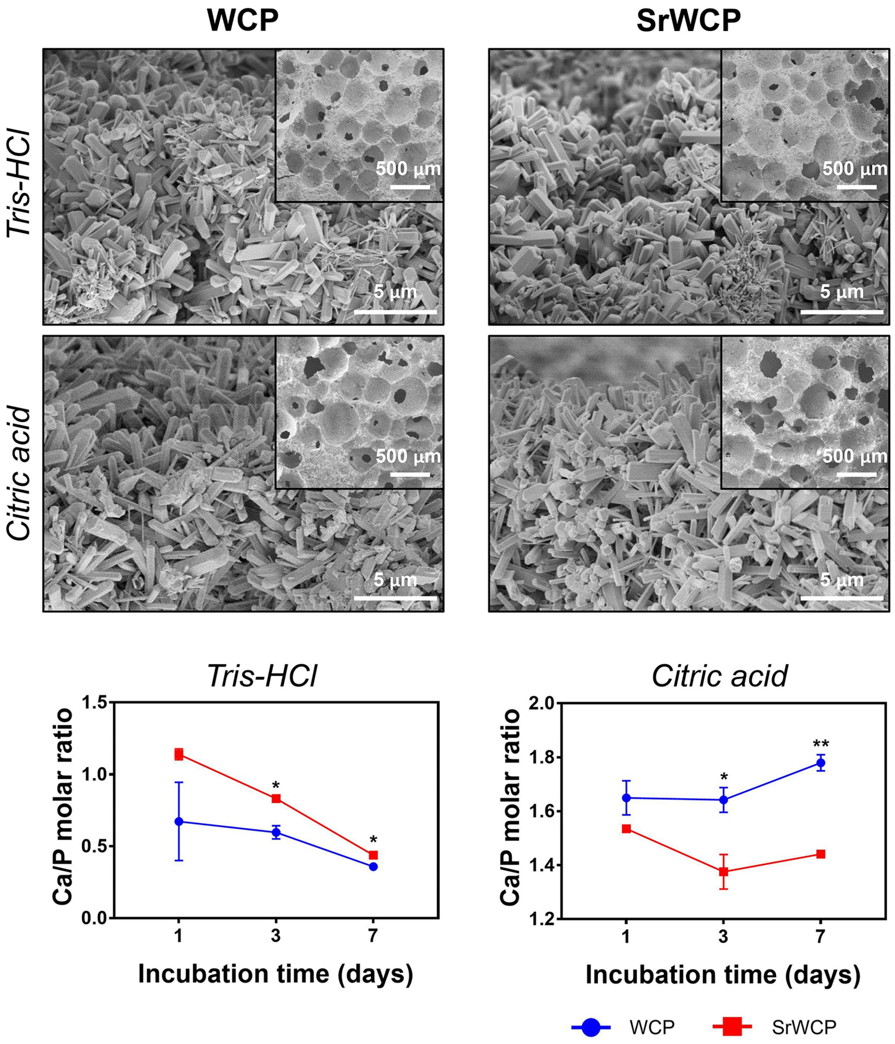

Figure S3. SEM images of the WCP and SrWCP bioceramics after immersion into Tris-HCL and citric acid solutions for 7 days and time-dependent changes of Ca/P molar ratio in Tris-HCL and citric acid solutions (*$p < 0.05$ vs the WCP group, **$p < 0.01$ vs the WCP group).

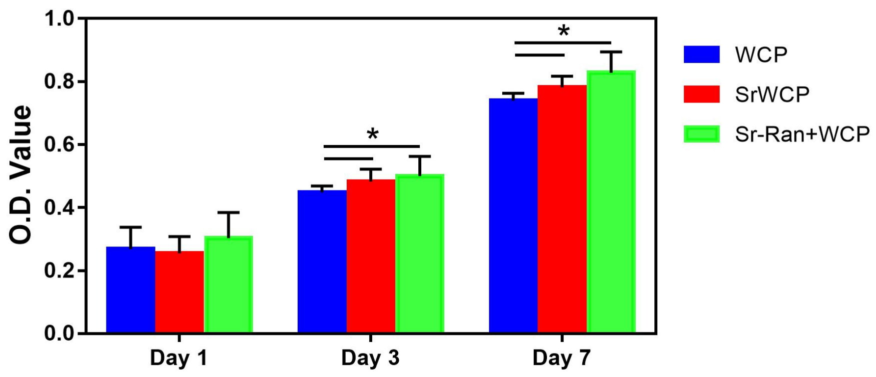

Figure S4. The MSCs grown on the different bioceramics quantified by CCK8 assay (*$p < 0.05$).

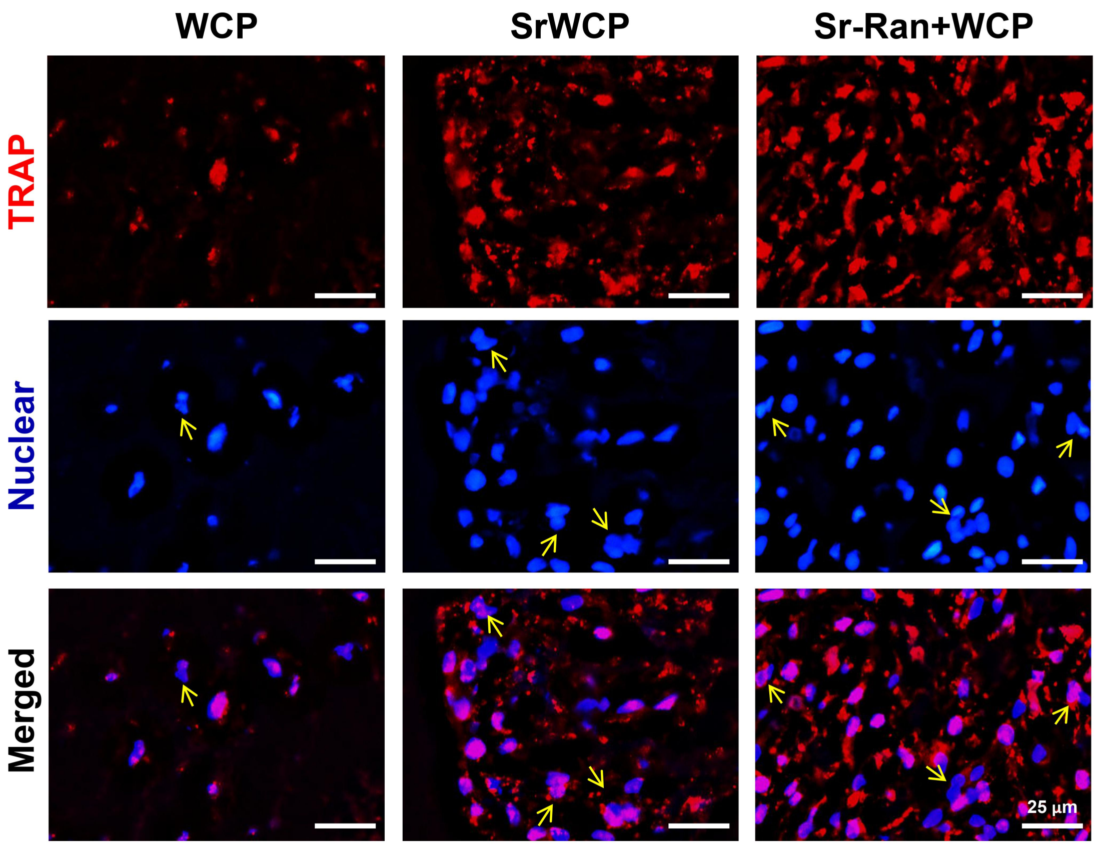

Figure S5. Immunofluorescence staining: red fluorescence indicates active TRAP; blue fluorescence indicates PI bound to the nuclei of cells. The yellow arrow represents the multinucleated osteoclast.

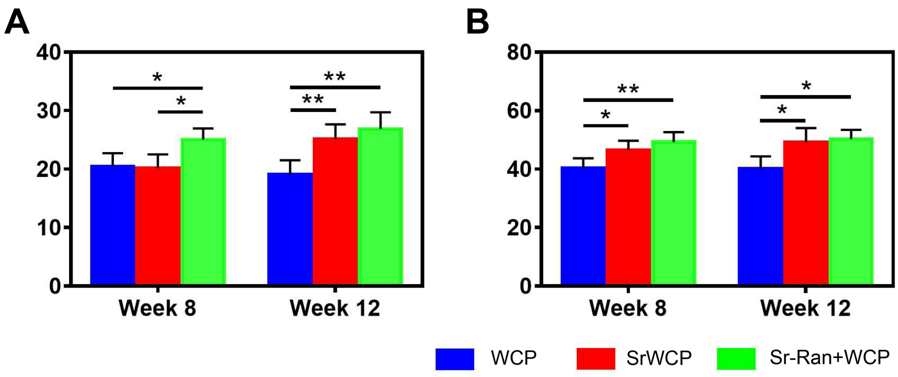

Figure S6. Quantitative analysis for the new bone area inside the drilled hole area (A) and gap area (B). At week 8, the Sr-Ran+WCP group showed higher new bone area inside the drilled hole area (p = 0.0479) and gap area (p = 0.0078) compared to the WCP group. Significant increase in the new bone area inside the drilled hole area (p = 0.0299) was observed in the SrWCP group compared to the Sr-Ran+WCP group at week 8. The WCP group also showed a significantly lower new bone area within the gap area than the SrWCP group (p = 0.0152) at week 8. At the end of week 12, the WCP group had a significantly lower new bone area inside the drilled hole area than both SrWCP (p = 0.0073) and Sr-Ran+WCP groups (p = 0.0018), and a significantly lower new bone area inside the gap area than the SrWCP (p = 0.0234) and Sr-Ran+WCP groups (p = 0.0161). No significant difference was observed between the SrWCP and Sr-Ran+WCP groups in the new bone area inside the drilled hole area and gap area at week 12.

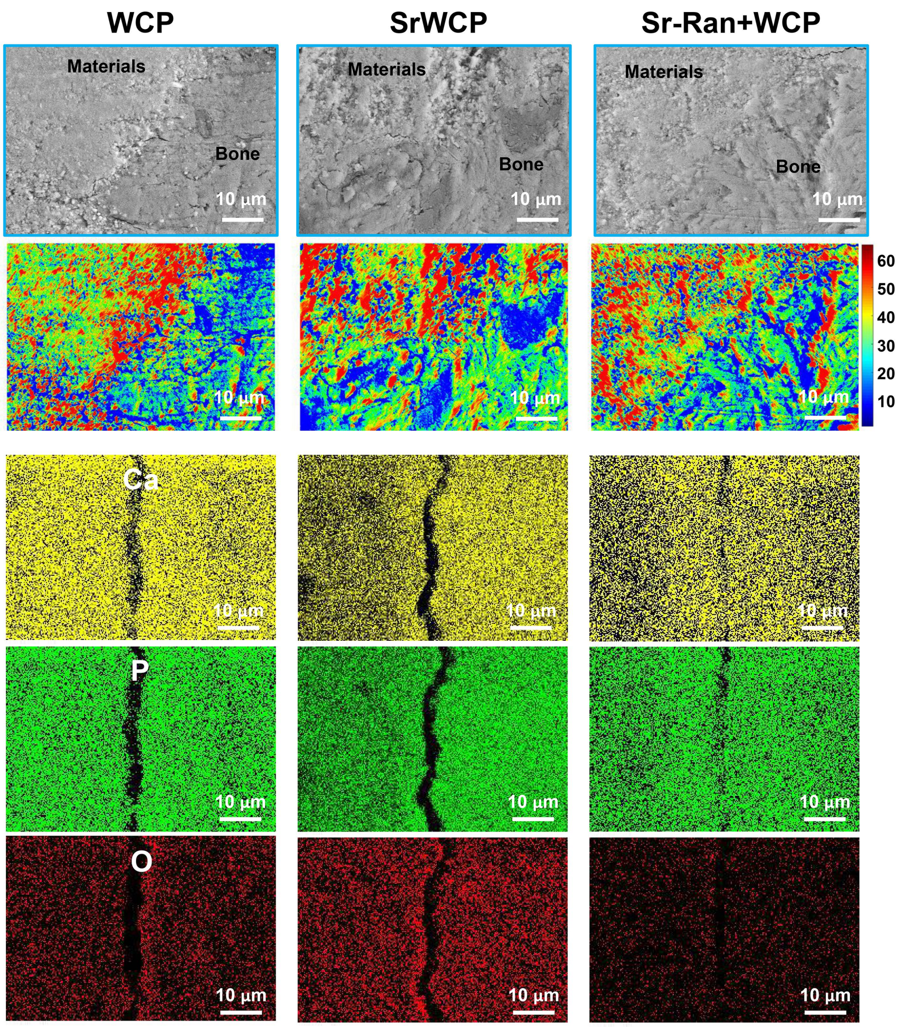

Figure S7. SEM observation and the respective pseudo-colored images of the unstained sections, and EDS mapping for the major elements (Ca, P and O) of the adjacent bone (Left) to the materials (Right) in the three groups.
